# Wheat Brefeldin-A ADP-Ribosylated Substrate TaANGUSTIFOLIA Negatively Regulates Cuticular Wax Biosynthesis

**DOI:** 10.3390/biom16101538

**Published:** 2026-10-21

**Authors:** Minawar Yusup, Haoyu Li, Pengfei Zhi, Jiao Liu, Cheng Chang

**Affiliations:** College of Life Sciences, Qingdao University, Qingdao 266071, China

**Keywords:** *Triticum aestivum* L., wax biosynthesis, transcriptional regulation, TaAN, TaMYB46, TaKCS1

## Abstract

Cuticular wax covers the epidermal surface of bread wheat (*Triticum aestivum* L.) and plays a key role in regulating wheat environmental adaptation. Elucidating the molecular mechanism underlying cuticular wax biosynthesis could facilitate wheat genetic improvement for stress resistance. Herein, we reveal that wheat brefeldin-A (BFA) ADP-ribosylated substrate TaANGUSTIFOLIA (TaAN) negatively regulates the biosynthesis of cuticular wax. Silencing of the *TaAN* gene in bread wheat resulted in significantly increased cuticular wax accumulation and decreased rates of water loss and chlorophyll leaching. Furthermore, we demonstrate that the TaAN protein directly suppresses transcription of the *TaMYB46* gene, and identify the wax biosynthesis gene *TaKCS1* as a target gene of the transcriptional activator TaMYB46. Moreover, we found that the *TaMYB46* and *TaKCS1* genes are epistatic to the *TaAN* gene in the regulation of cuticular wax biosynthesis. These findings elucidate that wheat BFA ADP-ribosylated substrate TaAN negatively regulates cuticular wax accumulation, probably via suppressing the TaMYB46-*TaKCS1* module, providing valuable information for wheat genetic improvement in cuticular wax-associated traits.

## 1. Introduction

A hydrophobic skin, the lipophilic cuticle, mainly composed of cutin matrices and wax mixtures, coats plant leaves, stems, flowers, and even roots during their primary growth [1,2,3,4,5]. The cuticle gets involved in plant developmental events and contributes to plant adaptation to drought, salinity, extreme temperatures, and even ultraviolet radiation [6,7,8]. The cereal crop bread wheat (*Triticum aestivum* L.) originated about 8500–9000 years ago, and is widely cultivated in order to provide dietary proteins and calories for humans [9]. Wheat yields and grain quality for food purposes have been seriously affected by abiotic and biotic stresses [10,11]. Uncovering the molecular mechanisms underlying wheat cuticular wax biosynthesis could contribute to wheat genetic improvement.

Wheat cuticular wax mixtures comprise very-long-chain (VLC, >C20) fatty acids and their derivatives, including alcohols, aldehydes, alkanes, alkyl esters, and even β-diketones in some tissues [12,13,14,15,16]. VLC wax mixtures are synthesized predominantly by modifying long-chain (C16 and C18) fatty acids in the endoplasmic reticulum (ER) [12,13,14,15,16]. Long-chain acyl-CoA synthetases (LACSs) firstly mediate the synthesis of C16 and C18 fatty acyl-CoAs, and fatty acid elongase (FAE) complex, composed of 3-ketoacyl-CoA synthase (KCS), 3-ketoacyl-CoA reductase (KCR), 3-hydroxyacyl-CoA dehydratase (HCD), and trans 2,3-enoyl-CoA reductase (ECR), subsequently catalyzes the elongation of C16 and C18 fatty acyl-CoAs to form VLC fatty acyl-CoAs [17,18,19,20,21]. The wax constituents, namely, VLC alcohols, aldehydes, fatty acids, alkanes, and alkyl esters, are ultimately modified from VLC fatty acyl-CoAs via either the alcohol-forming pathway or the alkane-forming pathway [22,23,24,25,26]. In bread wheat, cuticular wax biosynthesis is tightly regulated at the transcriptional level. For instance, wheat cuticular wax biosynthesis is activated by MYB-type transcription factors TaMYB30, TaMYB46, and TaMIXTA1/2 [27,28,29].

As an ancient protein, brefeldin-A (BFA) ADP-ribosylation substrate/C-terminal-binding protein-1 short-form (BARS/CtBP1-S) exists in a wide range of animals, such as flies, worms, and rats [30,31]. In the model plant *Arabidopsis thaliana*, AtANGUSTIFOLIA (AtAN) was identified as a single BARS/CtBP1-S protein. The *Arabidopsis AtAN* gene controls the polar elongation of leaf cells to regulate leaf width [32]. The Arabidopsis *an* mutant plant harboring a translocation or point mutation in the *AtAN* gene showed defects in the polarity-dependent elongation of leaf cells [32]. In addition to cell morphology modulation, AtAN plays a key role in the regulation of abiotic and biotic stress response [33]. The *an-t1* knockout mutant harboring a T-DNA insertion in the *AtAN* gene displayed increased resistance to drought stress, compared with the wild type [33]. However, the BFA-ADP-ribosylation substrate ANGUSTIFOLIA remains to be characterized in the important cereal crop bread wheat, especially in the regulation of cuticular wax biosynthesis.

In this study, we proposed the hypothesis that wheat BFA ADP-ribosylated substrate TaANGUSTIFOLIA (TaAN) might play a key role in the regulation of cuticular wax biosynthesis. To examine this hypothesis, we identified TaAN proteins from allohexaploid bread wheat and characterized its regulation of cuticular wax biosynthesis. We reveal that TaAN proteins directly suppress transcription of the *TaMYB46* gene encoding transcriptional activator of the cuticular wax biosynthesis gene *TaKCS1*, thereby inhibiting cuticular wax biosynthesis. These findings provide valuable information for wheat genetic improvement in sustainable agriculture.

## 2. Materials and Methods

### 2.1. Wheat Materials and Growth Conditions

Wheat cultivar Yannong999 was employed in this study. Wheat seedlings were cultivated in 300 mL pots containing mixtures of nutritive medium and sterile soil (1:1, *v*:*v*). Wheat plants were cultivated in a growth chamber under a 16 h light, 20 °C/8 h dark, 18 °C cycle with 150 μmol photons s^−1^ m^−2^ light intensity; irrigated with 60 mL of water per pot; and fertilized with water-soluble compound fertilizer Huawuque (Plant-Soul, Win-Tong, Shanghai, China).

### 2.2. Silencing of Wheat Genes

A barley stripe mosaic virus-induced gene silencing (BSMV-VIGS) assay was conducted as described to silence the *TaAN1*, *TaAN2*, or *TaMYB46* gene [27,28,29]. An approximately 200 bp fragment of the *TaAN1*, *TaAN2*, or *TaMYB46* gene was amplified using primers 5′AAGGAAGTTTAAAAGTTTGATTAGAGATGC3′/5′AACCACCACCACCGTAAAGAAGGAAATGAACTG3′, 5′AAGGAAGTTTAATAACTGTAAATAGACTAG3′/5′AACCACCACCACCGTCATCAGTGTTGTATTCTG3′, and 5′AAGGAAGTTTAAACTCAACTTGGAAATCAAGG3′/5′AACCACCACCACCGTAGTACTGCTGAGGGCACCAC3′ and cloned into the pCa-γbLIC vector. For the BSMV-VIGS assay, pCaBS-α, pCaBS-β, and pCa-γbLIC derivative constructs were transformed into *Agrobacterium tumefaciens* and agro-infiltrated into *Nicotiana benthamiana* leaves. After the appearance of virus symptoms, BSMV-infected *N. benthamiana* leaves were harvested and ground to inoculate seedlings of wheat cultivar Yannong 999. Newly grown wheat leaves with virus symptoms were harvested for subsequent experiments.

### 2.3. Analyses of Wheat Gene Transcription

Gene transcript accumulation levels and transcription rates were analyzed by Real-Time quantitative PCR (RT-qPCR) and nuclear Run-On assays, respectively, as previously described [27,28,29]. Total RNA was prepared from leaves of wheat BSMV-VIGS plants with silenced *TaAN1* (BSMV-*TaAN1*), *TaAN2* (BSMV-*TaAN2*), all endogenous *TaAN* (BSMV-*TaAN*), *TaMYB46* (BSMV-*TaMYB46*), or co-silenced *TaAN* and *TaMYB46* (BSMV-*TaAN* + BSMV-*TaMYB46*) genes using the RNAprep Pure Plant Kit (TIANGEN, Beijing, China) and subjected to the cDNA biosynthesis by TransScript IV, one-step gDNA removal and cDNA synthesis supermix (Transgen, Beijing, China) based on the kit instructions. Accumulation levels of *TaAN1, TaAN2*, *TaMYB46*, or *TaKCS1* gene transcripts were analyzed using gene-specific primer sequences 5′GTCGCCGGAGCGGGAGCT3′/5′CGCGGTCGGGGGAGCCGAG3′, 5′GGCCCGGCCCATTCCGTG3′/5′AAGGCGAGAGAGGTGAGG3′, 5′CAGGAAACAGCAATGTGG3′/5′GTTGACGATGAGGTCTTC3′, and 5′CCTTCATGGAGCGGGAGCG3′/5′ACGTGAGGCTGCCGCCGTG3′, and the geometric mean of two housekeeping genes, *TaACTIN* and *TaGADPH*, was used in the RT-qPCR analysis. These primers were designed after searching the wheat genomic sequence, to avoid potential off-target effects. For the nuclear run-on assay, wheat cell nuclei were extracted and used for the transcription reaction after being mixed with reaction buffer containing 25 mM biotin-16-UTP and 0.75 mM ATP, CTP, and GTP. After the RNA extraction, the biotin-labelled nascent RNA was harvested by streptavidin magnetic beads and subjected to RT-qPCR analysis.

### 2.4. Analyses of TaAN and TaMYB46 Enrichment at Gene Promoters

Enrichment of TaAN1-GFP and TaAN2-GFP at *TaMYB46* promoters and occupancy of TaMYB46-GFP on *TaKCS1* promoter regions were analyzed by chromatin immunoprecipitation–quantitative PCR (ChIP-qPCR) assay, as previously described [29]. Coding sequences of the *TaMYB46*, *TaAN1*, and *TaAN2* genes were amplified using the primers 5′GGGGACAAGTTTGTACAAAAAAGCAGGCTTCATGGCCCACTCGCCGGCCG3′/5′GGGGACCACTTTGTACAAGAAAGCTGGGTCATCTAGCCACCGGGTGACTC3′, 5′GGGGACAAGTTTGTACAAAAAAGCAGGCTTCATGTTGCGCGGCCCGGCCCA3′/5′GGGGACCACTTTGTACAAGAAAGCTGGGTCATCGAGCCACCTGGTGACG3′, and 5′GGGGACAAGTTTGTACAAAAAAGCAGGCTTCATGAGGAAGCCCGTGGAGTG3′/5′GGGGACCACTTTGTACAAGAAAGCTGGGTCCTCAACTTGGAAATCAAGGA3′ and cloned into the vector *pCAMBIA1300-GFP* to create the *TaMYB46-GFP*, *TaAN1-GFP*, and *TaAN2-GFP* constructs, using the GATEWAY cloning technology (Invitrogen, Waltham, MA, USA). An approximately 200 bp fragment of the *TaAN1*, *TaAN2*, and *TaMYB46* gene was amplified using primers 5′GGGGACAAGTTTGTACAAAAAAGCAGGCTTCAAGTTTGATTAGAGATGC3′/5′GGGGACCACTTTGTACAAGAAAGCTGGGTCAAAGAAGGAAATGAACTG3′, 5′GGGGACAAGTTTGTACAAAAAAGCAGGCTTCTAACTGTAAATAGACTAG3′/5′GGGGACCACTTTGTACAAGAAAGCTGGGTCCATCAGTGTTGTATTCTG3′, and 5′GGGGACAAGTTTGTACAAAAAAGCAGGCTTCCTCCGCCTACAGCTTCTTC3′/5′GGGGACCACTTTGTACAAGAAAGCTGGGTCCCGACCGTCAACAATGGCT3′ and cloned into the pIPKb007 vector to create the *RNAi-TaMYB46*, *RNAi-TaAN1*, and *RNAi-TaAN2* constructs, using the GATEWAY cloning technology (Invitrogen, Waltham, MA, USA). Wheat protoplast cells were prepared as described and transfected with *TaAN1-GFP*, *TaAN2-GFP*, or *TaMYB46-GFP* together with *RNAi-EV*, *RNAi-TaAN1*, *RNAi-TaAN2*, or *RNAi-TaMYB46* constructs. Antibody α-GFP (ab290, Abcam, Boston, MA, USA) was employed for the ChIP assay, and the qPCR analyses of *TaMYB46* and *TaKCS1* promoter fragments were performed using the primers 5′GGTCCTGGATGCAACGAC3′/5′TGCCATGGGAAGAGTGAT3′, 5′TCAAGAATTCAAAGACAA3′/5′CATGTCACATGCTTCTTA3′, 5′GAGAGCGTTGTTATGATC3′/5′AGCACCTCACTGCACTGA3′, 5′GGCTAATAGATAGCTTAC3′/5′CCTCACCGCCCCGATAAA3′,5′AGGCACTAATGGTAGTAG3′/5′TTGCGAGAGAGGAATTGA3′, 5′GGTTCTTTAGATCCATTG3′/5′TTTGGCCGAAAATGGCTA3′, 5′TTGAGCAGCTAGTTTGATC3′/5′CGCTCATTCGGTCTATTCC3′, 5′GAAAGAGACGGGTAGTAGA3′/5′AGGCTGCCGCTGCCGCTGC3′, 5′GAGATAAGCCCTATAAACC3′/5′TATAGGGCTTATCTCAAAA3′, 5′GTGGATAGTAGACAGGTAG3′/5′GTTGGATCGCTCGAGGCTG3′, 5′GTGTTACTTACATATTGAC3′/5′ GTGTGGTGTTATTGGACGT3′, and 5′GACCGAGGCGCCGGAACCG3′/5′GTGTGCGCCTTGCAGTTTG3′. DNA recovery from the ChIP assay was expressed as a percentage of the input, and a fragment of the intergenic region was employed as the negative control. Wheat protoplast cells expressing indicated RNAi vectors were included as another negative control.

### 2.5. Analysis of Cuticular Wax Accumulation

Accumulation of wheat cuticular wax was quantified by gas chromatography–mass spectrometry (GC-MS) assays as previously described [27,28,29]. Cuticular wax mixtures were extracted from leaves of wheat BSMV-VIGS plants with silenced *TaAN1* (BSMV-*TaAN1*), *TaAN2* (BSMV-*TaAN2*), all endogenous *TaAN* (BSMV-*TaAN*), *TaMYB46* (BSMV-*TaMYB46*), or co-silenced *TaAN* and *TaMYB46* (BSMV-*TaAN* + BSMV-*TaMYB46*) genes, using chloroform (Merck), and n-Tetracosane (Merck, Rahway, NJ, USA) was added as the internal standard. Cuticular wax extracts were dried under N_2_ gas and analyzed using a capillary GC (5890 Series II, Agilent Technologies, Santa Clara, CA, USA) and a flame ionization detector (6890N, Agilent Technologies, Santa Clara, CA, USA), in addition to a mass spectrometer (MSD5973, Agilent Technologies, Santa Clara, CA, USA). Wax compositions were identified and quantified based on the retention times and flame ionization detector peak areas.

### 2.6. Analysis of Wheat Leaf Cuticle Permeability

Wheat leaf cuticle permeability was analyzed by water loss and chlorophyll leaching assays, as described [29]. Wheat BSMV-VIGS plants with silenced *TaAN1* (BSMV-*TaAN1*), *TaAN2* (BSMV-*TaAN2*), or all endogenous *TaAN* (BSMV-*TaAN*) genes were dipped in ultrapure water in the dark to maintain stomatal closure, as described [29], and the wheat leaves were detached. Detached leaves were weighed hourly for 12 h to measure the water loss rate, as described, and chlorophyll was extracted from detached leaves with 80% ethanol and measured hourly for 12 h to analyze the chlorophyll leaching rate, as previously reported.

### 2.7. Statistical Analysis

At least 5 wheat leaves (for the analyses of gene expression, cuticular wax, and SA accumulation measurements, and leaf cuticle permeability) and 300 mg wheat protoplasts (for the analysis of protein enrichment at gene promoters) were randomly chosen for each group and statistically analyzed in one experiment. Shapiro–Wilk, Kolmogorov–Smirnov, Levene, or Bartlett tests were performed to confirm normal distribution and homogeneity of variance. For the analysis of the statistical significance of differences between two groups, data were analyzed using Student’s *t*-test, and the values represent the mean ± standard deviation (n.s., *p* > 0.05; * 0.01 < *p* < 0.05; ** *p* < 0.01; n.s. indicates no significant difference). For the analysis of the statistical significance of differences among three or more groups, data were analyzed using one-way ANOVA with Duncan’s post hoc test (bars with different letters are significantly different, *p* < 0.05). Each assay was repeated with three biological replicates, using separately prepared samples with similar results.

## 3. Results

### 3.1. Identification of Wheat Brefeldin-A ADP-Ribosylated Substrate TaANGUSTIFOLIA (TaAN)

**Figure 1:**
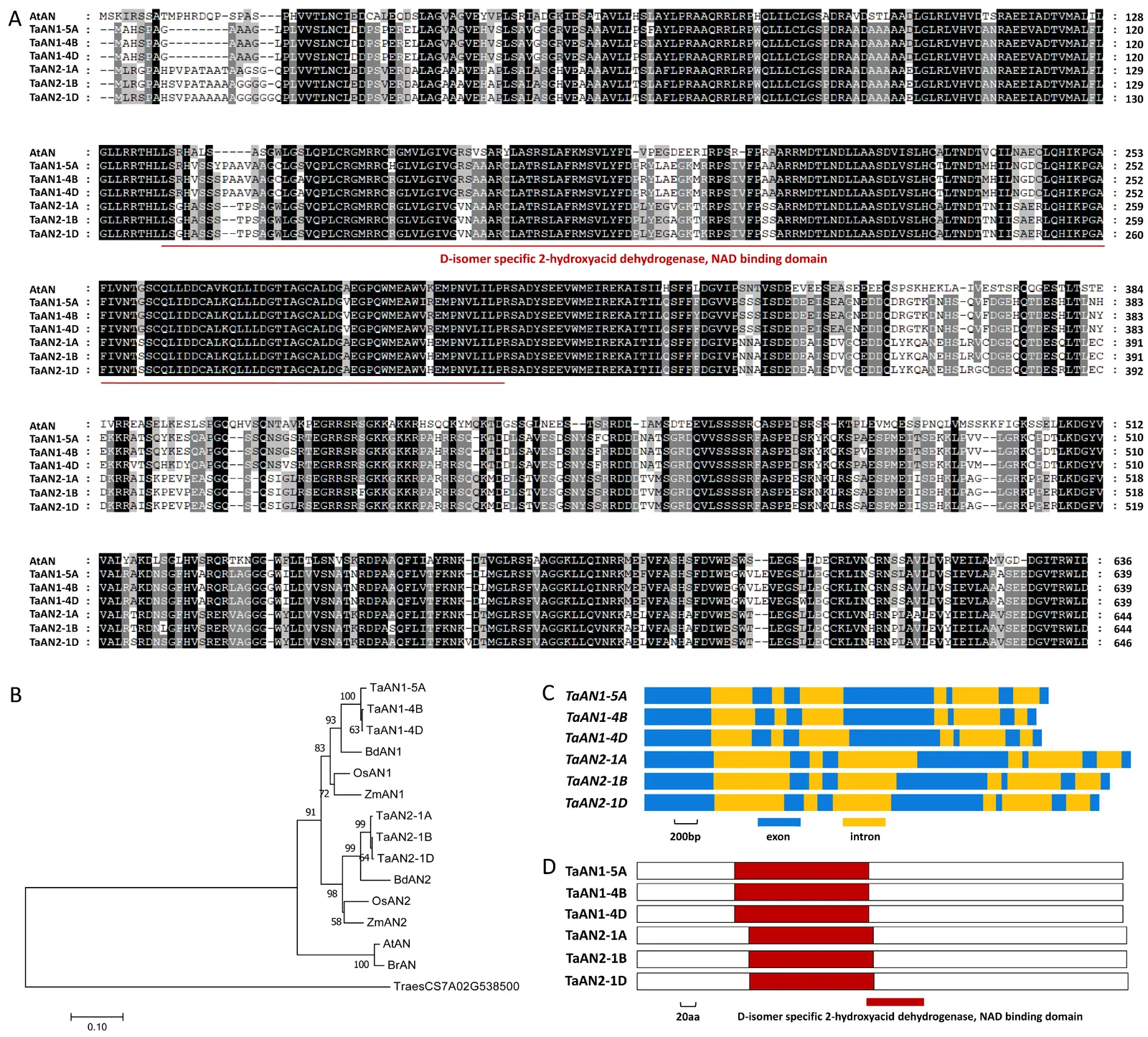
Homology-based identification of wheat brefeldin-A ADP-ribosylated substrate TaAN. (**A**) Sequence comparison of wheat TaAN1 and TaAN2 and Arabidopsis AtAN proteins. Identical residues among the 7 protein sequences are in dark shading, while residues conserved among the 4 protein sequences are shaded in gray. The D-isomer-specific 2-hydroxyacid dehydrogenase, NAD-binding (2-Hacid_dh_C) domain is underlined. (**B**) Phylogenetic analysis of wheat TaAN1, TaAN2, *Arabidopsis* AtAN, Brassica BrAN, rice OsAN1, OsAN2, *Brachypodium* BdAN1, BdAN2, maize ZmAN1, and ZmAN2 proteins. Scale bar = amino acid substitutions per site. (**C**) Exon and intron arrangement of the *TaAN* genomic sequences. (**D**) 2-Hacid_dh_C domain arrangement of TaAN protein sequence.

To identify the wheat brefeldin-A ADP-ribosylated substrate ANGUSTIFOLIA (AN), we searched the wheat reference genome using the Arabidopsis AtAN (At1g01510) protein sequence as a query, and identified TaAN1 and TaAN2. Three *TaAN1* genes, located on wheat chromosomes 5A, 4B, and 4D, were designated as *TaAN1-5A* (*TraesCS5A02G493200*), *TaAN1-4B* (*TraesCS4B02G319000*), and *TaAN1-4D* (*TraesCS4D02G315500*). Similarly, three *TaAN2* genes, located on wheat chromosomes 1A, 1B, and 1D, were designated as *TaAN2-1A* (*TraesCS1A02G185300*), *TaAN2-1B* (*TraesCS1B02G193200*), and *TaAN2-1D* (*TraesCS1D02G191600*). Sequence comparison indicated that wheat TaAN1-5A, TaAN1-4B, TaAN1-4D, TaAN2-1A, TaAN2-1B, and TaAN2-1D proteins shared above 61% amino acid identity with the Arabidopsis AtAN protein (Figure 1A). As shown in Figure 1B, phylogenetic analysis confirmed that wheat TaAN1-5A, TaAN1-4B, TaAN1-4D, TaAN2-1A, TaAN2-1B, and TaAN2-1D proteins are close homologs of *Arabidopsis* AtAN, *Brassica* BrAN, rice OsAN1, OsAN2, *Brachypodium* BdAN1, BdAN2, maize ZmAN1, and ZmAN2. As shown in Figure 1C, seven exons and six introns were identified from genomic sequences of allelic *TaAN1-5A*, *TaAN1-4B*, *TaAN1-4D*, *TaAN2-1A*, *TaAN2-1B*, and *TaAN2-1D* genes. A conserved D-isomer specific 2-hydroxyacid dehydrogenase, NAD binding (2-Hacid_dh_C) domain was identified from the TaAN1-5A, TaAN1-4B, TaAN1-4D, TaAN2-1A, TaAN2-1B, and TaAN2-1D proteins (Figure 1D).

### 3.2. Functional Characterization of TaAN in the Regulation of Wheat Cuticular Wax Biosynthesis

**Figure 2:**
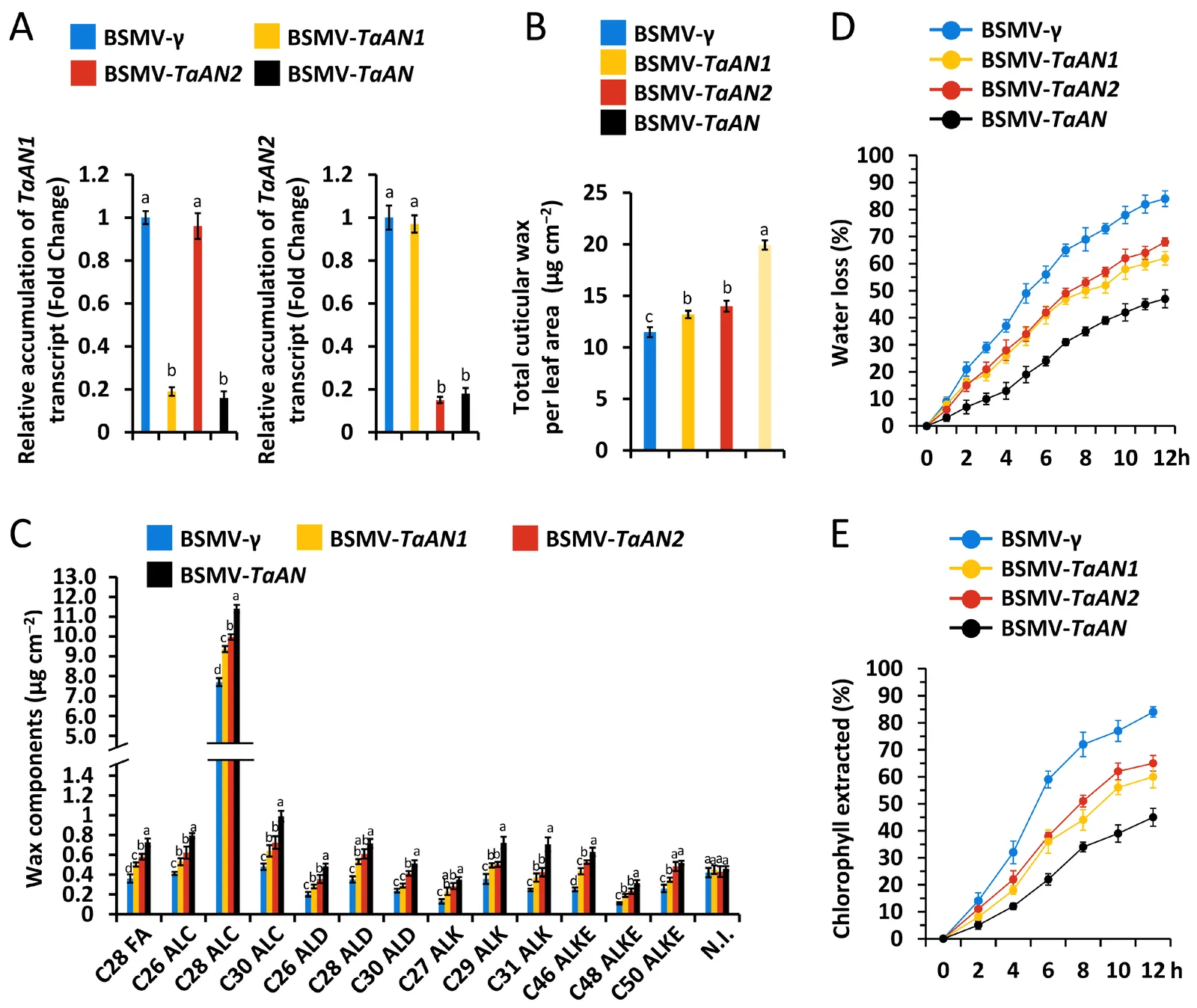
Characterization of cuticular wax accumulation in wheat plants with silenced *TaAN* genes. (**A**) RT-qPCR analysis of *TaAN1* and *TaAN2* gene transcript accumulation levels in the wheat leaves with *TaAN1* (BSMV-*TaAN1*), *TaAN2* (BSMV-*TaAN2*), or all endogenous *TaAN* (BSMV-*TaAN*) genes silenced. Amounts of total cuticular wax (**B**) and major cuticular wax components (**C**) in wheat leaves with *TaAN1*, *TaAN2*, or all endogenous *TaAN* genes silenced. FA, fatty acid; ALC, alcohol; ALD, aldehyde; ALK, alkane; ALKE, alkyl ester; N.I., not-identified compound. Water loss rates (**D**) and chlorophyll leaching ratios (**E**) were analyzed in the wheat leaves with *TaAN1*, *TaAN2*, or all endogenous *TaAN* genes silenced. Leaves of wheat plants infected with the BSMV-γ empty vector were employed as the negative control. Data were statistically analyzed by one-way ANOVA with Duncan’s post hoc test, and different letters above error bars represent statistical significance (*p* < 0.05).

To examine the potential regulation of wheat *TaAN* genes on cuticular wax biosynthesis, we separately silenced the endogenous *TaAN1* or *TaAN2* genes by performing the barley stripe mosaic virus-induced gene silencing (BSMV-VIGS) assay in the wheat cultivar Yannong 999. The RT-qPCR assay demonstrated that the transcript accumulation levels of *TaAN1* or *TaAN2* genes decreased significantly in wheat leaves with *TaAN1*, *TaAN2*, or all endogenous *TaAN* genes silenced (Figure 2A). As shown in Figure 2B, silencing of the endogenous *TaAN1* (BSMV-*TaAN1*) or *TaAN2* (BSMV-*TaAN2*) gene resulted in a significant increase in cuticular wax accumulation, while simultaneously silencing all endogenous *TaAN* (BSMV-*TaAN1* + BSMV-*TaAN2*) genes resulted in a further increase in cuticular wax accumulation, compared with the BSMV-γ control. Cuticular wax composition analyses revealed that wax alcohols (ALCs), alkanes (ALKs), fatty acids (FAs), aldehydes (ALDs), and alkyl esters (ALKEs) showed varying degrees of increase in the wheat leaves with silenced *TaAN1*, *TaAN2*, or all endogenous *TaAN* genes, compared with the BSMV-γ control (Figure 2C). Excised-leaf water loss and chlorophyll leaching assays were conducted to examine the potential regulation of wheat *TaAN* genes on leaf cuticle permeability. As shown in Figure 2D,E, silencing of all endogenous *TaAN1* or *TaAN2* genes resulted in reduced rates of water loss and chlorophyll leaching, whereas simultaneously silencing all endogenous *TaAN* genes resulted in further reductions of water loss and chlorophyll leaching rates.

### 3.3. Analysis of TaAN Regulation on TaMYB46 Gene Transcription

**Figure 3:**
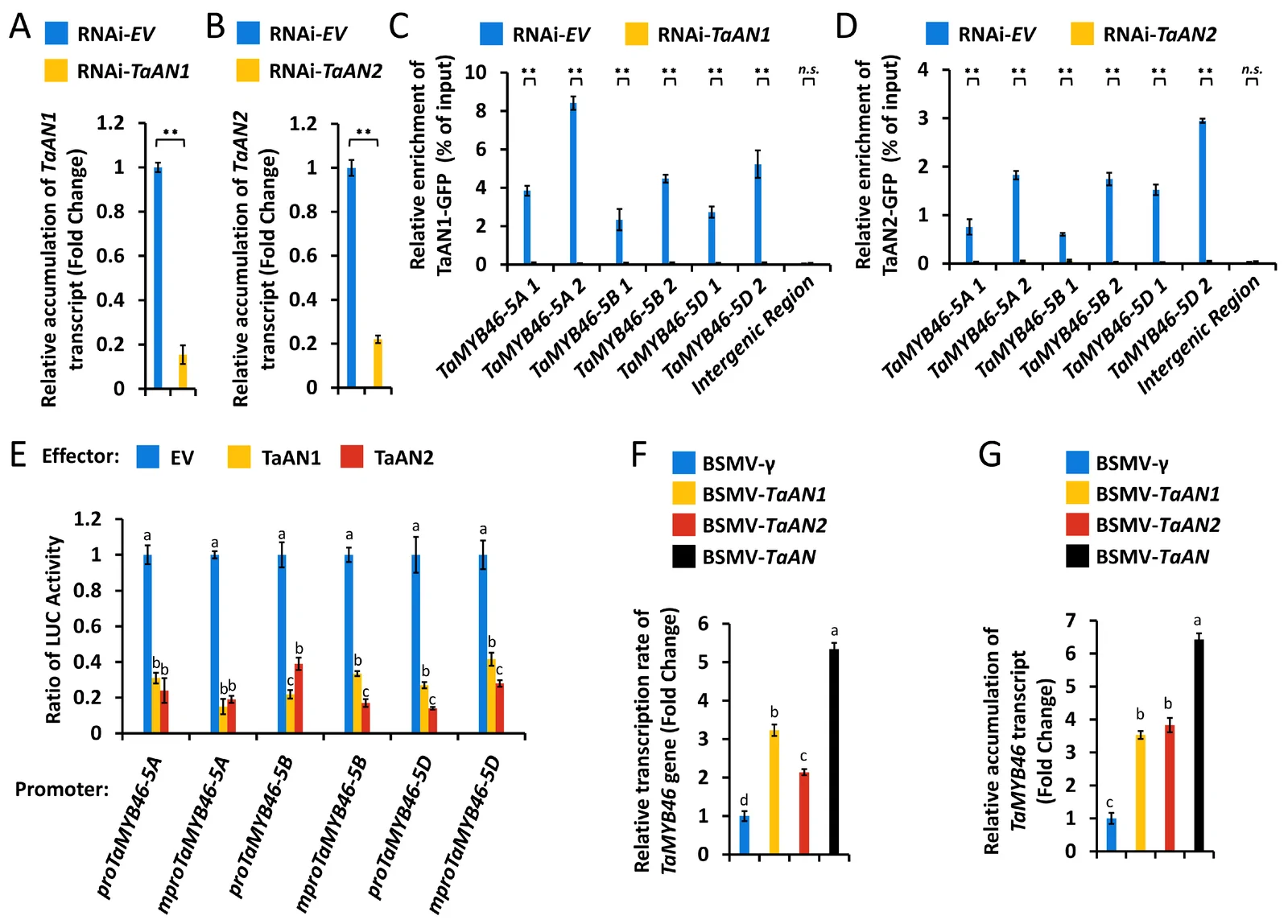
Analysis of transcriptional regulation of wheat BFA ADP-ribosylated substrate TaAN on the *TaMYB46* gene. RT-qPCR analysis of *TaAN1* (**A**) and *TaAN2* (**B**) gene transcript accumulation levels in wheat protoplast cells with *TaAN1* (RNAi-*TaAN1*) or *TaAN2* (RNAi-*TaAN2*) gene silenced. ChIP-qPCR analysis of TaAN1-GFP (**C**) and TaAN2-GFP (**D**) enrichment at the *TaMYB46* gene promoter regions in wheat protoplast cells co-transfected with constructs *TaAN1-GFP* or *TaAN2-GFP* together with *RNAi-EV*, *RNAi-TaAN1*, or *RNAi-TaAN2*. A fragment of the intergenic region was employed as the negative control, and wheat protoplast cells expressing the indicated RNAi vectors were included as another negative control. (**E**) Transrepression of the *TaMYB46* gene promoter by TaAN1 and TaAN2 in the wheat protoplast cells transfected with the indicated pairs of effectors and LUC reporter. The LUC activity obtained from wheat protoplasts expressing DBD empty effectors (negative control) was set to 1.0. Renilla LUC was employed as an internal control. (**F**) Nuclear run-on analysis of *TaMYB46* gene transcription rate in the wheat leaves with *TaAN1* (BSMV-*TaAN1*), *TaAN2* (BSMV-*TaAN2*), or all endogenous *TaAN* (BSMV-*TaAN*) genes silenced. (**G**) RT-qPCR analysis of *TaMYB46* gene transcript accumulation levels in the wheat leaves with *TaAN1*, *TaAN2*, or all endogenous *TaAN* genes silenced. Data were statistically analyzed either by Student’s *t*-test (for (**A**–**D**), ** *p* < 0.01; n.s. indicates not significant (*p* ≥ 0.05)) or by one-way ANOVA with Duncan’s post hoc test (for (**E**–**G**), different letters above error bars represent statistical significance, *p* < 0.05).

To examine the potential enrichment of wheat brefeldin-A ADP-ribosylated substrates TaAN1 and TaAN2 at *TaMYB46* promoter regions, we expressed TaAN1-GFP or TaAN2-GFP fusion protein in the wheat protoplast cells and conducted the ChIP-qPCR assay. An intergenic region was employed as the negative control, and wheat protoplast cells expressing the indicated RNAi vectors were included as another negative control. An RT-qPCR assay demonstrated that the transcript accumulation levels of the *TaAN1* or *TaAN2* gene decreased significantly in wheat protoplast cells with the *TaAN1* or *TaAN2* gene silenced (Figure 3A,B). As shown in Figure 3C,D, fragments of *TaMYB46* promoters were co-immunoprecipitated with the α-GFP antibody against TaAN1-GFP and TaAN2-GFP, indicating that wheat BFA ADP-ribosylated substrates TaAN1 and TaAN2 enrich at the *TaMYB46* promoter regions. To examine whether TaAN could modulate gene transcription driven by *TaMYB46* promoters, we conducted a wheat leaf protoplast transfection assay. The ratio of LUC activity driven by the *TaMYB46* promoter decreased from 1, for Gal4 DNA-binding domain (DBD) control groups, to below 0.42, for TaAN1 or TaAN2 overaccumulation groups (Figure 3E). Nuclear run-on and RT-qPCR assays demonstrated that *TaMYB46* gene transcription rates and transcript accumulation levels were significantly increased in wheat leaves with silenced *TaAN1* or *TaAN2* genes, while simultaneously silencing all endogenous *TaAN* genes resulted in a further increase in *TaMYB46* gene transcription rates and transcript accumulation levels (Figure 3F,G).

### 3.4. Analysis of TaMYB46 Regulation on Transcription of TaKCS1 Gene

**Figure 4:**
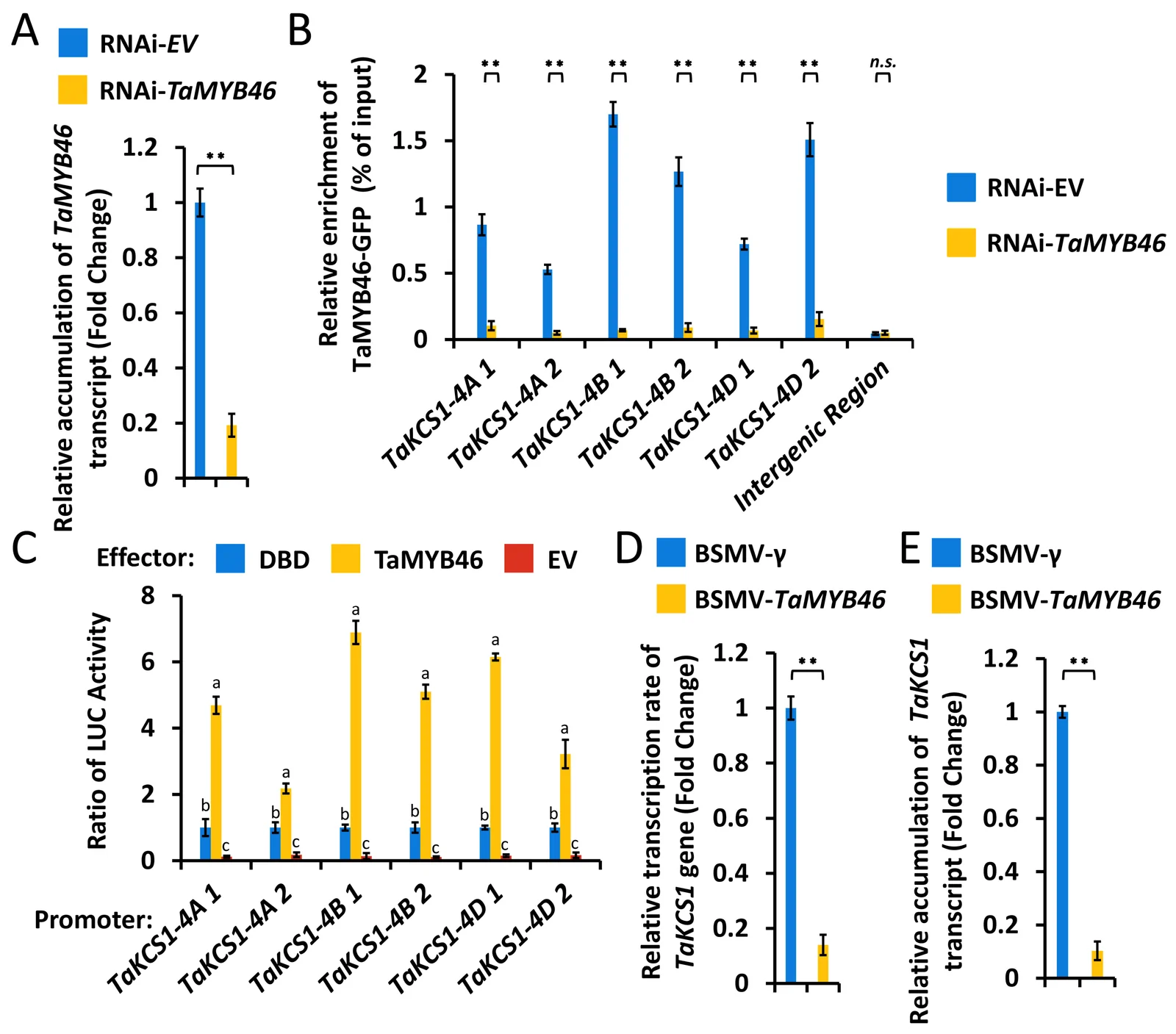
Analysis of transcriptional regulation of the wheat transcription factor TaMYB46 on *TaKCS1* gene. (**A**) RT-qPCR analysis of *TaMYB46* gene transcript accumulation levels in wheat protoplast cells with silenced *TaMYB46* (RNAi-*TaMYB46*) gene. (**B**) ChIP-qPCR analysis of TaMYB46-GFP enrichment at *TaKCS1* gene promoter regions in wheat protoplast cells co-transfected with *TaMYB46-GFP* constructs together with *RNAi-TaMYB46*. A fragment of the intergenic region was employed as the negative control, and wheat protoplast cells expressing the indicated RNAi vectors were included as another negative control. (**C**) Transactivation of *TaKCS1* gene promoters by TaMYB46 in the wheat protoplast cells transfected with the indicated pairs of effectors and the LUC reporter. The LUC activity obtained from wheat protoplasts expressing DBD empty effectors (negative control) was set to 1.0. (**D**) Nuclear run-on and RT-qPCR analysis of *TaKCS1* gene transcription rate and transcript accumulation levels in wheat leaves with the *TaMYB46* gene silenced. Data were statistically analyzed either by Student’s *t*-test (for (**B**–**E**), ** *p* < 0.01; n.s. indicates not significant (*p* ≥ 0.05)) or by one-way ANOVA with Duncan’s post hoc test (for (**C**), different letters above error bars represent statistical significance, *p* < 0.05).

To examine the potential enrichment of wheat transcription factor TaMYB46 at promoter regions of *TaKCS1*, the homologous gene of Arabidopsis *AtKCS1*, we expressed the TaMYB46-GFP fusion protein in wheat protoplast cells and conducted the ChIP-qPCR assay. An intergenic region was employed as the negative control, and wheat protoplast cells expressing the indicated RNAi vectors were included as another negative control. An RT-qPCR assay demonstrated that the transcript accumulation levels of the *TaMYB46* gene decreased significantly in wheat protoplast cells silencing the *TaMYB46* gene (Figure 4A). As shown in Figure 4B, fragments of *TaKCS1* gene promoters were co-immunoprecipitated with the α-GFP antibody against TaMYB46-GFP, indicating that the wheat transcription factor TaMYB46 occupies the *TaKCS1* promoter regions. To examine whether TaMYB46 could modulate gene transcription driven by *TaKCS1* gene promoters, we conducted the wheat leaf protoplast transfection assay. The ratio of LUC activity driven by the *TaMYB46* promoter increased from 1 for Gal4 DNA-binding domain (DBD) control groups to above 6.9 for TaMYB46 overaccumulation groups (Figure 4C). Nuclear run-on and RT-qPCR assays demonstrated that transcription rates and transcript accumulation levels of the *TaKCS1* gene were significantly reduced in wheat leaves with the *TaMYB46* gene silenced (Figure 4D,E).

### 3.5. Characterization of Wheat Cuticular Wax Biosynthesis Governed by the TaAN-TaMYB46-TaKCS1 Module

**Figure 5:**
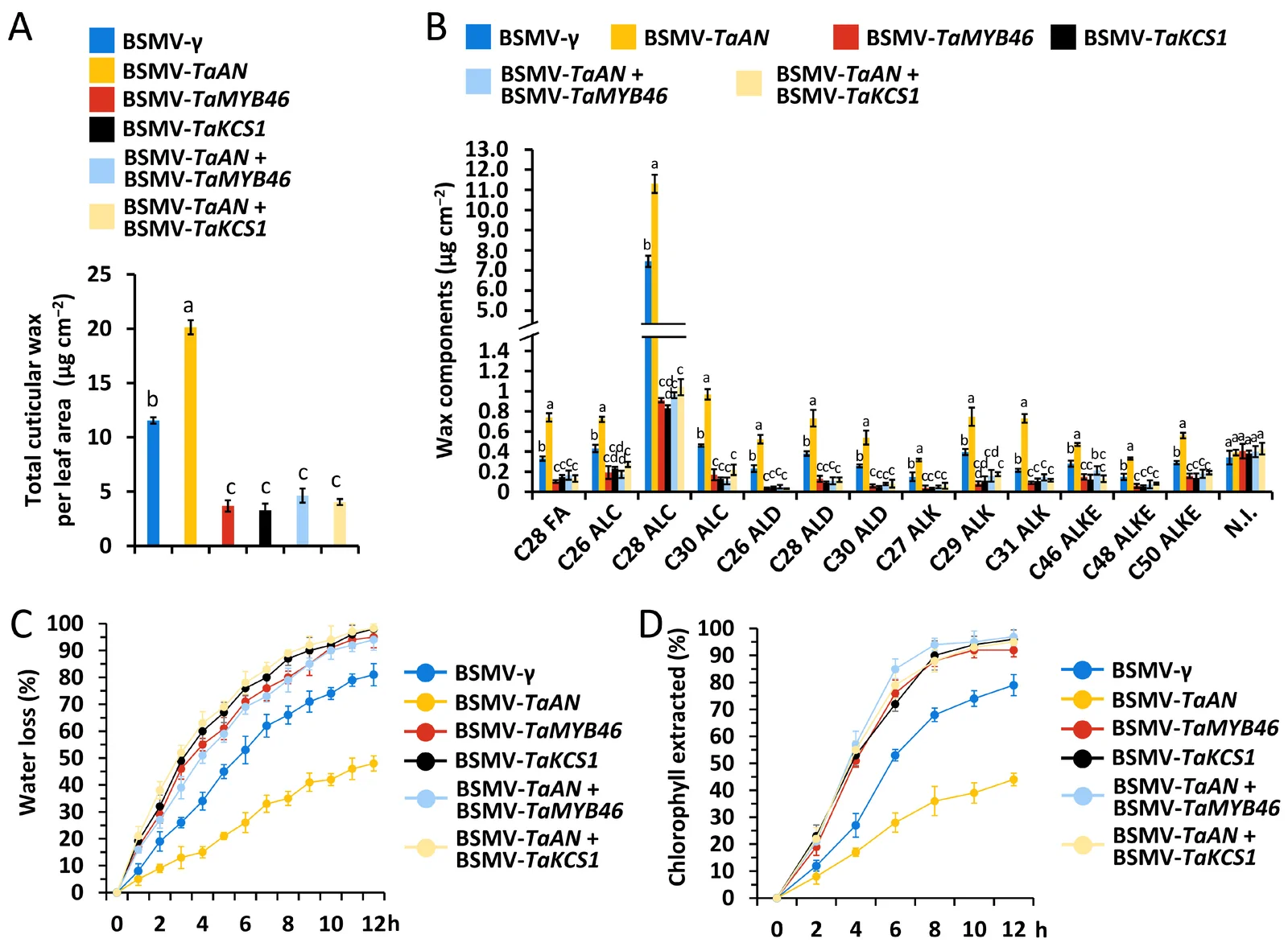
Characterization of cuticular wax accumulation in wheat plants with silenced *TaAN* and *TaMYB46 or TaKCS1* genes. Amounts of total cuticular wax (**A**) and major cuticular wax components (**B**) in the wheat leaves with *TaAN* (BSMV-*TaAN*), *TaMYB46* (BSMV-*TaMYB46*), *TaKCS1* (BSMV-*TaKCS1*), or *TaAN* and *TaMYB46* (BSMV-*TaAN* + BSMV-*TaMYB46*), *TaAN* and *TaKCS1* (BSMV-*TaAN* + BSMV-*TaKCS1*) genes silenced. Water loss rates (**C**) and chlorophyll leaching ratios (**D**) were analyzed in the wheat leaves with *TaAN*, *TaMYB46*, *TaKCS1*, or *TaAN* and *TaMYB46, TaAN* and *TaKCS1* genes silenced. Data were statistically analyzed by one-way ANOVA with Duncan’s post hoc test, and different letters above error bars represent statistical significance (*p* < 0.05).

To examine the contribution of wheat transcription factor gene *TaMYB46* and wax biosynthesis gene *TaKCS1* to the suppression of BFA ADP-ribosylated substrate TaAN on cuticular wax accumulation, we simultaneously silenced *TaAN* and *TaMYB46* or *TaAN* and *TaKCS1* by BSMV-VIGS, and analyzed the cuticular wax accumulation in these BSMV-VIGS leaves. As shown in Figure 5A, accumulation levels of total cuticular wax increased to above 171% in the *TaAN*-silencing wheat leaves, but decreased to below 41% in the *TaMYB46*-silencing, *TaKCS1*-silencing, *TaAN/TaMYB46*-cosilencing, or *TaAN/TaKCS1*-cosilencing wheat leaves. Wax alcohol, aldehyde, alkane, fatty acid, and alkyl ester accumulation levels increased significantly in *TaAN*-silencing wheat leaves but decreased markedly in *TaMYB46*-silencing, *TaKCS1*-silencing, *TaAN*/*TaMYB46*-cosilencing, or *TaAN*/*TaKCS1*-cosilencing wheat leaves (Figure 5B). Excised-leaf water loss and chlorophyll leaching assays were conducted to examine the leaf cuticle permeability on these wheat leaves with *TaAN*-silenced, *TaMYB46*-silenced, *TaKCS1*-silenced, *TaAN*/*TaMYB46*-cosilenced, or *TaAN*/*TaKCS1*-cosilenced. As shown in Figure 5C,D, significantly reduced rates of water loss and chlorophyll leaching were observed on the wheat leaves with *TaAN*-silenced, while silencing of *TaMYB46*, *TaKCS1*, co-silencing of *TaAN* and *TaMYB46,* and co-silencing of *TaAN* and *TaKCS1* genes resulted in remarkably enhanced water loss and chlorophyll leaching rates, compared with those of BSMV-γ control wheat leaves.

## 4. Discussion

### 4.1. Wheat Transcription Factor TaMYB46 Activates Transcription of Wax Biosynthesis Gene TaKCS1

It was recently reported that transcription factor TaMYB46 directly activates the cuticular lipid biosynthesis gene *TaLACS1* [29]. Silencing of *TaMYB46* resulted in the reduction of cuticular wax loads to 29.2%, while silencing of the *TaLACS1* gene led to the reduction of cuticular wax accumulation to 65.5%, suggesting that TaMYB46 might activate other cuticular wax biosynthesis genes [29]. In this study, ChIP-qPCR and dual-luciferase reporter assays demonstrated that TaMYB46 protein enriches at the *TaKCS1* promoter regions and activates transcription driven by the *TaKCS1* gene promoters. Nuclear run-on and RT-qPCR assays demonstrated that *TaKCS1* transcription rates and transcript accumulation levels were significantly reduced in wheat leaves with the *TaMYB46* gene silenced, suggesting that TaMYB46 directly activates *TaKCS1* genes. Ketoacyl-CoA synthase TaKCS1 functions as an essential component of wax biosynthetic machinery in bread wheat [27]. Silencing of the *TaKCS1* gene by BSMV-VIGS resulted in a reduction of cuticular wax loads to 24.7% and increased leaf cuticle permeability [27]. These studies indicated that wheat transcription factor TaMYB46 transcriptionally activates both the *TaKCS1* and *TaLACS1* genes. Arabidopsis transcription factor AtMYB46 was reported to directly regulate the expression of *AtLACS1*, *AtKCS1*, and *AtCER1* genes to stimulate cuticular wax biosynthesis [34]. These studies suggested that transcriptional activation of cuticular wax biosynthesis genes *LACS1* and *KCS1* by transcription factor MYB46 might be conserved among the dicot model plant *A. thaliana* and the monocot cereal plant bread wheat.

In bread wheat, transcription of the ketoacyl-CoA synthase gene *TaKCS1* was activated by transcription factors TaMYB30 and TaMIXTA1/2 [27,28]. Herein, we demonstrated that transcription factor TaMYB46 transcriptionally activates the *TaKCS1* gene as well. Increasing evidence revealed that cuticular wax accumulations and compositions vary among plant tissues and developmental stages, and plant cuticular wax biosynthesis is tightly regulated by environmental and developmental cues. Exploring the differential regulation of transcription factors TaMYB46, TaMYB30, and TaMIXTA1/2 on the *TaKCS1* gene at the spatiotemporal and environmental levels could shed novel insight into the complexity of wheat wax biosynthesis in future research.

### 4.2. Wheat Brefeldin-A ADP-Ribosylated Substrates TaAN1 and TaAN2 Partially Redundantly Suppress TaMYB46 Transcription

As an evolutionarily conserved protein, BFA-ADP-ribosylation substrate/C-terminal-binding protein-1 short-form (BARS/CtBP1-S) exists widely in animals and plants [30,31]. The Arabidopsis *AtAN* gene regulates leaf development and plant response to abiotic and biotic stresses [32,33]. However, the function of BFA-ADP-ribosylation substrate remains unknown in monocot plants. In this study, we identified six *TaAN1* and *TaAN2* genes located on wheat chromosomes 5A, 4B, 4D, 1A, 1B, and 1D. Surprisingly, no *AN* homolog was found on chromosome 4A. ChIP-qPCR and dual-luciferase reporter assays demonstrated that TaAN1 and TaAN2 proteins enrich at the *TaMYB46* promoters and suppress the transcription driven by the *TaMYB46* gene promoters. Nuclear run-on and RT-qPCR assays demonstrated that *TaMYB46* transcription rates and transcript accumulation levels were significantly increased in wheat leaves with silenced *TaAN1* or *TaAN2* genes, while simultaneously silencing all endogenous *TaAN1* and *TaAN2* genes resulted in a further increase in *TaMYB46* gene transcription rates and transcript accumulation levels, suggesting that BFA-ADP-ribosylated substrates TaAN1 and TaAN2 partially redundantly repress *TaMYB46* transcription.

However, many scientific questions remain to be answered. For instance, animal BFA-ADP-ribosylation substrates function as transcriptional co-repressors by recruiting epigenetic modulators [30,31]. Whether and how BFA ADP-ribosylated substrates TaANs employ similar epigenetic mechanisms to inhibit *TaMYB46* transcription remains to be determined. Previous studies have revealed that wheat histone deacetylase TaHDA19 and Chromatin Assembly Factor-1 (CAF-1), composed of FASCIATA1 (TaFAS1), FASCIATA2 (TaFAS2), and MULTIPLE SUPPRESSOR OF IRA1 (TaMSI1), function as epigenetic suppressors of cuticular wax biosynthesis [35,36]. Exploring the potential interaction of brefeldin-A ADP-ribosylated substrates TaANs with histone deacetylase TaHDA19, CAF-1 components TaFAS1, TaFAS2, and TaMSI1 might shed novel insight into the suppression mechanism of TaANs in the regulation of *TaMYB46* gene transcription.

### 4.3. Wheat TaAN-TaMYB46-TaKCS1 Pathway Governs Cuticular Wax Biosynthesis

Functional analysis revealed that wheat BFA ADP-ribosylated substrates TaAN1 and TaAN2 partially redundantly suppress cuticular wax. In contrast, wheat transcription factor TaMYB46 activates transcription of the ketoacyl-CoA synthase gene *TaKCS1* and positively regulates cuticular wax biosynthesis. In this study, we performed the co-silencing assay to analyze the potential genetic relationship of wheat *TaANs* genes with *TaMYB46* and *TaKCS1* genes in the regulation of cuticular wax biosynthesis. Total cuticular wax increased to above 171% in the *TaAN*-silencing wheat leaves, but decreased to below 41% in the *TaMYB46*-silencing, *TaKCS1*-silencing, *TaAN/TaMYB46*-cosilencing, or *TaAN/TaKCS1*-cosilencing wheat leaves. This analysis indicated that silencing the *TaMYB46* and *TaKCS1* genes is epistatic to silencing the *TaAN* gene in the regualtion of wheat cuticular wax biosynthesis, suggesting that *TaMYB46* and *TaKCS1* genes function downstream of *TaAN* gene in controlling cuticular wax biosynthesis. These genetic epistatic results imply that wheat BFA-ADP-ribosylation substrate TaAN negatively regulates wheat cuticular wax biosynthesis, probably by transcriptionally inhibiting *TaMYB46* transcription.

In plant cuticular wax biosynthesis, long-chain acyl-CoA synthetases LACSs mediate the synthesis of C16 and C18 fatty acyl-CoAs, whereas 3-ketoacyl-CoA reductases KCSs function in the fatty acid elongase complex to catalyze the elongation of C16 and C18 fatty acyl-CoAs to form VLC fatty acyl-CoAs, the important precursors for the biosynthesis of wax components like VLC fatty acids, alcohols, aldehydes, alkanes, and alkyl esters [16,17,18]. Herein, the quantification of wax components revealed that accumulation levels of wax alcohols, aldehydes, alkanes, fatty acids, and alkyl esters displayed significant increases in the *TaAN*-silencing wheat leaves but showed remarkable reductions in the *TaMYB46*-silencing, *TaKCS1*-silencing, *TaAN*/*TaMYB46*-cosilencing, and *TaAN*/*TaKCS1*-cosilencing wheat leaves, further confirming that the wheat TaAN-TaMYB46-TaKCS1 pathway governs cuticular wax biosynthesis, probably by controlling the synthesis of important precursors VLC fatty acyl-CoAs.

In this study, we characterized BFA ADP-ribosylated substrate TaAN and transcription factor TaMYB46 as key regulators of cuticular wax. Increasing evidence revealed that cuticular wax greatly contributes to plant resistance against environmental stresses like water deficit, salinity, unfavorable temperatures, and even ultraviolet radiation [8]. Genetically manipulating *TaAN* and *TaMYB46* genes by TILLING and advanced genome editing techniques like CRISPR-Cas might contribute to wheat improvement for cuticular wax-associated traits and stress resistance [37,38,39,40]. In addition, large-scale genotype analyses of *TaAN* and *TaMYB46* genes in global wheat cultivars and identifying potential elite haplotypes of these genes might contribute to precise wheat breeding for improved cuticular wax-associated traits [41,42].

## 5. Conclusions

In this study, we revealed that wheat BFA ADP-ribosylated substrate TaAN negatively regulates biosynthesis of cuticular wax. Knockdown of *TaAN* gene expression in bread wheat led to reduced cuticular wax accumulation. Furthermore, we demonstrated that the TaAN proteins directly suppress transcription of the *TaMYB46* gene and identified the wax biosynthesis gene *TaKCS1* as a target gene of the transcriptional activator TaMYB46. In addition, we found that *TaMYB46* and *TaKCS1* genes function downstream of the *TaAN* gene in the regulation of cuticular wax biosynthesis. These findings elucidated that wheat BFA ADP-ribosylated substrate TaAN negatively regulates cuticular wax accumulation, probably via suppressing the TaMYB46-*TaKCS1* module, providing valuable information for improvement of cuticular wax-associated traits in bread wheat, in the interests of sustainable agriculture.

## Data Availability

The data presented here are available upon request by means of correspondence.
